# Small Intestinal Permeability and Gut-Transit Time Determined with Low and High Molecular Weight Fluorescein Isothiocyanate-Dextrans in C3H Mice

**DOI:** 10.3390/nu10060685

**Published:** 2018-05-28

**Authors:** Anni Woting, Michael Blaut

**Affiliations:** Department of Gastrointestinal Microbiology, German Institute of Human Nutrition Potsdam-Rehbruecke, Arthur-Scheunert-Allee 114-116, 14558 Nuthetal, Germany; blaut@dife.de

**Keywords:** intestinal permeability, FITC dextran, transit time

## Abstract

Protocols for intestinal permeability measurements in mice using 4-kDa fluorescein isothiocyanate-conjugated (FITC) dextran differ considerably among laboratories on the blood-sampling time. To find the optimal point in time for blood sampling, we administered 4-kDa FITC dextran to C3H mice and monitored the marker in plasma over 8 h. We also determined gut-transit time using 70-kDa FITC dextran, which does not cross the intestinal epithelium. The 4-kDa FITC dextran concentration in plasma reached its maximum 45 min after administration. The 70-kDa FITC dextran reached the jejunum after 15 min and passed the entire small intestine within 1 h after its administration, demonstrating that 4-kDa FITC dextran measured in plasma 1 h after its oral application is a marker of small intestinal permeability.

## 1. Introduction

The epithelial layer lining the intestinal tract represents a barrier that protects the host against harmful substances, prevents microbes from entering the body, and thereby contributes to maintaining host integrity. However, increased intestinal permeability and subsequent metabolic endotoxemia are hallmarks of obesity and type-2 diabetes in both humans and mice [1]. Hence, the relationship between gut barrier integrity and metabolic diseases has been investigated comprehensively by measuring gut permeability using different marker molecules such as non-digestible 4-kDa dextran conjugated with fluorescein isothiocyanate (FITC dextran) [2,3]. Once orally administered, this fluorescent marker transits the gastrointestinal tract and passively crosses the intestinal epithelium. The concentration of 4-kDa FITC dextran in plasma, which can be determined easily with a fluorimeter, represents a measure for paracellular permeability of the intestinal epithelium. The gut-transit time affects the speed of movement of the 4-kDa FITC dextran along the digestive tract. Hence, the time required for the marker to reach a given gut section and the time elapsed between the oral application of 4-kDa FITC dextran, and the point when the marker reaches its maximal concentration in the blood denote the segment in which it crossed the epithelium. However, the duration reported for 4-kDa FITC dextran to reach its maximal concentration in plasma varies and therefore the transit time of 4-kDa FITC dextran and the site of its uptake in the gut appear to differ among different laboratories for the same mouse strain. A recent study in adult wildtype C57BL/6 mice reported that 4-kDa FITC dextran reaches its maximal concentration in plasma within the first 2 h after oral application [4]. Assuming a physiologic gut-transit time of 5 to 8 h [5,6], uptake of the marker in the aforementioned mouse study probably occurred in the small intestine. In contrast to Cifarelli et al. [4], others reported that 4-kDa FITC dextran reached its maximal concentration in the plasma of adult wildtype C57BL/6 mice 4 h after its oral application [7]. With a physiologic gut-transit time, FITC dextran can reach the colon within 4 h. Hence, one might assume that the 4-kDa FITC dextran concentration detected in plasma after this period of time reflected colonic permeability. These examples illustrate that the time required for 4-kDa FITC dextran to reach its maximal concentration in plasma differs among studies. Hence, if the peak concentration in plasma and the gut-transit time of this marker are unknown, but the 4-kDa FITC dextran concentration in plasma is measured at only one point in time, the location of gut barrier dysfunction cannot be assessed accurately.

For our work on metabolic syndrome and gut barrier we have used C3H/HeOu mice, which represent a mouse model of moderate obesity [8,9]. To our knowledge, the time elapsed between oral application and maximal concentration of 4-kDa FITC dextran in plasma as well as gut-transit time of this marker have not experimentally been determined for this mouse model. It is not known, where in the gut the 4-kDa FITC dextran is taken up and whether this marker determines small intestinal or rather colonic permeability in C3H/HeOu mice.

In this short report, we systematically analyzed the time elapsed between oral application and occurrence of 4-kDa FITC dextran in plasma of C3H/HeOu mice. In parallel, we determined the accurate gut-transit time in another set of mice using 70-kDa FITC dextran, which does not cross the epithelium. Our results demonstrate that the uptake of 4-kDa FITC dextran in C3H/HeOu mice mainly occurred in duodenum and jejunum. It may be concluded that the 4-kDa FITC dextran assay in C3H/HeOu mice measures permeability in these two small intestinal segments.

## 2. Materials and Methods

### 2.1. Animals

Specific-pathogen-free male C3H/HeOuJ mice were obtained from the animal facility of the German Institute of Human Nutrition, Potsdam-Rehbruecke, Germany. The mice were maintained under a 12-h light-dark cycle in polycarbonate cages on wood chips at 22 ± 2 °C and 55 ± 5% air humidity. All mice had ad libitum access to standard chow (Altromin 1314; Altromin, Lage, Germany) and water.

### 2.2. Experimental Setup

The experiment was approved by the Animal Welfare Committee of the State of Brandenburg (Approval no. V3-2347-10-2011). The 12-week-old male mice were fasted for 6 h and 4-kDa FITC dextran (Sigma-Aldrich, 600 mg/kg body weight, 80 mg/mL phosphate-buffered saline, PBS: NaCl, 8.0 g/L; KCl, 0.2 g/L; Na_2_HPO_4_, 1.44 g/L; KH_2_PO_4_, 0.24 g/L; pH 7.4) or 70-kDa FITC dextran (100 µL, 5 mg/mL PBS) was applied orally at a single dose. Three mice each were anaesthetized after 30 min, 1, 1.5, 2 and 8 h; another three mice were anaesthetized after 15 and 45 min and blood from the retrobulbar capillary plexus was sampled into heparinized tubes for 4-kDa FITC-dextran analyses. Plasma was obtained after centrifugation at 2000× *g* for 5 min. The intestinal concentration of 4-kDa FITC dextran was determined in the mice after they had been killed, namely 45 min and 8 h after oral application, as described for 70-kDa FITC dextran. The intestinal concentration of 70-kDa FITC dextran was determined after 0, 15, 30, 45 min and after 1, 1.5, 2, 3, 8 h. Three mice per point in time were killed. The gastrointestinal tracts were removed and placed on ice-cold glass plates. The small intestine was evenly cut into 5 segments and the colon was cut into 2 segments. Each segment was placed in 1 mL PBS. Stomach and caecum were placed in 5 mL PBS. Tissues were chopped, the liberated luminal contents were homogenized for 45 s and tissue and coarse particles were removed by centrifugation (300× *g*, 3 min). The supernatant was used for measuring the markers. Another group of three mice were treated with PBS and their plasma and gut contents served as blank.

### 2.3. Fluorescent Measurement

Plasma was diluted 1:5 (*v*/*v*) in PBS. Gut contents were diluted 1:10 or 1:100 (*v*/*v*) with PBS for the 70-kDa or 4-kDa FITC dextran analyses, respectively. Fluorescence was measured spectrophotometrically (Infinite M200 PRO, Tecan, Crailsheim, Germany) in 96-well plates (excitation: 485 nm, emission: 528 nm). FITC dextran concentrations were calculated with the help of standard concentrations prepared in PBS ranging from 0 to 250 µg/mL 4-kDa FITC dextran or 0 to 1250 µg/mL 70-kDa FITC dextran. Emission signals in plasma and gut contents of the mice receiving PBS were subtracted from those of mice treated with the 4-kDa or the 70-kDa FITC dextran. The fluorescence signal of luminal 70-kDa FITC dextran in each segment was related to the sum of the fluorescence signals in all segments of the gastrointestinal tract.

## 3. Results and Discussion

The measurement of gut permeability with 4-kDa FITC dextran is a useful method for studying the link between endotoxemia and metabolic syndrome. However, to our knowledge, it has not experimentally been demonstrated where exactly in the gut of C3H/HeOu mice the 4-kDa FITC dextran crosses the epithelium and hence, for which gut segment this fluorescent marker indicates permeability.

### 3.1. Monitoring 4-kDa FITC Dextran in Plasma over 8 Hours after Oral Application

**Figure 1:**
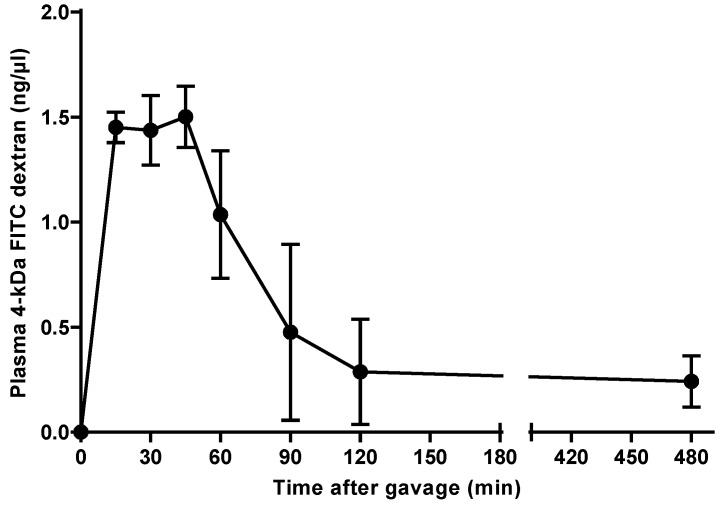
Concentration of 4-kDa fluorescein isothiocyanate (FITC) dextran in plasma of C3H mice. The fluorescent marker was administered orally and its concentration was monitored over 8 h. Mean ± SEM, *n* = 3.

To determine the site of 4-kDa FITC dextran uptake in the intestinal tract of C3H/HeOu mice, we monitored its concentration in plasma of one set of mice. Fifteen minutes after gavage, the 4-kDa FITC dextran became detectable in the plasma of the mice and reached its maximal concentration 45 min after its administration (1.50 ± 0.15 ng/µL) and thereafter started to decrease (Figure 1). A relatively low concentration of 0.24 ± 0.12 ng/µL 4-kDa FITC dextran in plasma was still detectable after 8 h.

The time required for 4-kDa FITC dextran to reach its maximal level in plasma of C57BL/6 mice was reported to be less than 2 or 4 h [4,7], with the latter being considerably longer than the 45 min observed in our C3H/HeOu mice. These large differences may have been caused by factors that influenced intestinal uptake and renal excretion of this marker. FITC dextrans are excreted through glomerular filtration [10,11], but there is no literature on their elimination in C57BL/6 and C3H/HeOu mice. The quantity of 4-kDa FITC dextran reaching the blood may be affected by the presence of intestinal contents, which may dilute or adsorb the marker in the gut. Moreover, the degree of filling of the intestine can modify peristalsis with possible consequences for the site of uptake of 4-kDa FITC dextran. To avoid such interferences, mice are usually fasted prior to the measurement. After fasting our C3H/HeOu mice for 6 h, the concentration of 4-kDa FITC dextran in plasma peaked at 45 min after oral application. Similarly, Cani et al. [12] also fasted their mice for 6 h and these mice displayed a higher 4-kDa FITC dextran concentration in plasma 1 h after gavage than 4 h after gavage. In contrast, the C57BL/6 mice employed by Cifarelli et al. [4] were fasted for 12 h, but similar to our study and that of Cani et al. [12], the 4-kDa FITC dextran peak in plasma was detected within 2 h after application. This comparison suggests that the duration of fasting is not a major factor that influences the appearance of this marker in plasma. However, the proportion of 4-kDa FITC dextran reaching the blood may be affected by fasting.

Mouse strain-dependent differences in intestinal permeability may also have affected the uptake of 4-kDa FITC dextran. It was reported that C57BL/6J mice display a more than twofold higher intestinal permeability for 4-kDa FITC dextran compared with A/J mice [13], whereas C57BL/6J and BALB/cJ mice do not differ in this regard [3]. However, intestinal permeability for 4-kDa FITC dextran has never been compared directly between C57BL/6 mice and C3H/HeOu mice. Another reason why the time for 4-kDa FITC dextran to reach its maximal concentration in the plasma differed between our male C3H/HeOu mice and those of Cifarelli et al. [4] and Patel et al. [7] might be gender, as these researchers used both male and female C57BL/6 mice. Estrogen maintains the function of the intestinal barrier by promoting the secretion of bicarbonate into the gut and by enhancing the expression of tight-junction proteins [14]. Hence, it is conceivable that the female mice included in the studies of Cifarelli et al. and Patel et al. had a reduced gut permeability compared to our male C3H/HeOu mice. This possibly explains the late appearance of the maximal 4-kDa FITC dextran concentration in plasma reported by Patel at al. compared with our study (4 h vs 45 min after oral application, respectively). However, such an effect was not observed by Cifarelli et al. [4], who reported the plasma 4-kDa FITC dextran levels to be maximal 2 h after oral application, even though they also included female mice in their study. This argues against estrogen being responsible for the observed differences, but does not exclude it. Unfortunately, Cifarelli et al. and Patel et al. did not report the ratio of male and female mice.

Gut-microbiota composition might be another reason for the discrepancies among the reported intestinal sites of uptake and the periods of time required for 4-kDa FITC dextran to be taken up in C57BL/6 mice and our C3H/HeOu mice. The vendors’ microbial environment and hygiene regimen, the sources of the chow ingredients, and the types of caging and bedding in animal facilities may influence the microbiota composition and thereby impede reproducibility [15,16]. Therefore, microbiota-induced variations in immune responses and metabolic parameters may have affected the permeability of the intestinal epithelium for 4-kDa FITC dextran in the C57BL/6 mice [7] and in our C3H/HeOu mice. For example, pathogens such as *Helicobacter pylori* increase epithelial permeability, whereas probiotics, such as *Lactobacillus plantarum,* reduce it [17]. A recent study demonstrated that the microbiota of aged mice, and ageing as such, increase intestinal permeability [18]. Whether certain bacterial species, a shift in bacterial metabolism, or changes in microbe-microbe interactions affected the intestinal barrier was not investigated in that study. Anyway, we do not have any indications that in our experiments intestinal permeability for 4-kDa FITC dextran was affected by certain bacteria or by certain age-related compositional changes in the microbiota.

### 3.2. Gut-Transit Time Determined with 70-kDa FITC Dextran

**Figure 2:**
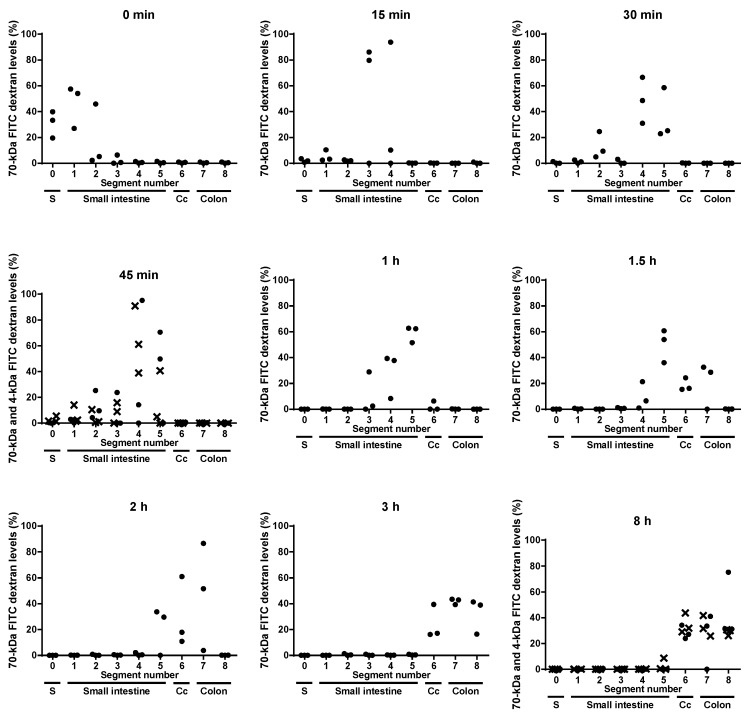
Levels of 70-kDa FITC dextran (●) and 4-kDa FITC dextran (**×**) in luminal contents of stomach (S), five small intestinal segments, caecum (Cc) and two colonic segments measured directly after gavage (0 min) and 15, 30 and 45 min and 1, 1.5, 2, 3 and 8 h after gavage. Each dot represents one mouse.

Aim of this study was to identify the gut segment in C3H/HeOu mice, in which 4-kDa FITC dextran transits into the blood stream. The 4-kDa FITC dextran was detectable in jejunum (segments 3 and 4) and ileum (segment 5) 45 min and in cecum and colon 8 h after oral application (Figure 2). However, the passage of the 4-kDa FITC dextran from the intestine into the circulation led to a dilution of the marker in the intestinal contents and consequently in an imprecise quantification of this marker therein and hence in an inaccurate measurement of the intestinal transit time. Therefore, the accurate gut-transit time was determined using a marker that does not cross the epithelium, 70-kDa FITC dextran. Both markers, 4-kDa and 70-kDa FITC dextran, were detected in jejunum and ileum as well as in cecum and colon, 45 min and 8 h after application, respectively (Figure 2). Even though the exact quantification of 4-kDa FITC dextran owing to its uptake was impeded, the results indicate that the 4-kDa FITC dextran and the 70-kDa FITC dextran moved with the same speed through the intestinal tract.

Fifteen minutes after its oral application, the 70-kDa FITC dextran reached the jejunum (segments 3 and 4) (Figure 2). Within this short period of time, the 4-kDa FITC dextran almost reached its peak concentration in plasma (Figure 1). Given that 4-kDa and 70-kDa FITC dextran have the same gut-transit time and that the 70-kDa FITC dextran passed the duodenum (segments 1 and 2) and reached the jejunum (segments 3 and 4) within 15 min after its administration, it may be concluded that the 4-kDa FITC dextran was preferentially taken up in the duodenum and the jejunum.

One hour after gavage, i.e., when the plasma concentration of 4-kDa FITC dextran started to decrease (Figure 1), the 70-kDa FITC dextran reached the ileum in all mice (Figure 2). Hence, the 4-kDa FITC dextran detected in the plasma of C3H/HeOu mice 1 h after its application reflects small intestinal permeability.

The 70-kDa FITC dextran reached the cecum and the colon 1.5 h after it had orally been applied and remained detectable in the colons of the mice for up to 8 h after application (Figure 2). This suggests that the detection of 4-kDa FITC dextran in plasma more than 1.5 h after its oral application represents colonic rather than small intestinal permeability. However, it cannot be ruled out that the marker molecules taken up in the small intestine still circulated in the blood because of a low glomerular filtration rate or that 4-kDa FITC dextran had been taken up in the colon after 1.5 h. Therefore, more specific measurements for the in vivo determination of colonic permeability in C3H/HeOu mice could be used, such as multi-sugar permeability tests. For instance, the recovery in urine of orally applied lactulose, which escapes digestion in the small intestine, is considered a marker for small intestinal permeability. The recovery of orally administered non-fermentable sucralose in urine indicates whole intestinal permeability. The 24-h lactulose excretion subtracted from the 24-h sucralose excretion represents a measurement of colonic paracellular permeability [17]. These sugar tests are extensive, but a useful alternative to 4-kDa FITC dextran for the measurement of colonic permeability in C3H/HeOu mice.

While the maximal concentration of 4-kDa FITC dextran in plasma of C3H/HeOu mice was detected within 1.5 h after administration of the marker, a delayed peak concentration or a second peak concentration of 4-kDa FITC dextran in plasma later than 1.5 h, as it might occur in other mouse strains such as C57BL/6 mice, would undoubtedly indicate colonic permeability, provided that these mice display a physiologic gut-transit time of 5 to 8 h [5,6]. Indeed, several research groups measured the concentration of this marker in plasma 4 h after gavage to determine colonic permeability in C57BL/6 mice. Of note is that the measurement of colonic permeability in C57BL/6 mice with the concentration of 4-kDa FITC dextran in plasma 4 h after its administration would require that the small intestinal epithelium be less permeable to 4-kDa FITC dextran than the colonic epithelium so that the majority of the marker would pass into the colon. This contradicts our finding that the small intestinal epithelium in C3H/HeOu mice is very permeable to 4-kDa FITC dextran transit and emphasizes the importance of determining, by time-dependent sampling of plasma and measurement of the gut-transit time, in which gut section the 4-kDa FITC dextran uptake occurs. Furthermore, our data were generated in adult, healthy, male C3H/HeOu mice fed a standard chow. The gut-transit time for 4-kDa FITC dextran and the time course of the concentration of this marker in plasma might be different in mice suffering from diet-induced obesity, diabetes or non-alcoholic fatty liver disease because diets rich in fat or sugars like fructose alter intestinal motility [1,17]. Besides diet, age, gender, microbial status and mouse genetics may also affect the intestinal site of 4-kDa FITC dextran uptake and this should be considered in gut permeability measurements.

In summary, 4-kDa FITC dextran in C3H/HeOu mice rapidly crosses the epithelium of the duodenum and the jejunum. Epithelial permeability of the small intestine can be assessed in these mice by measuring plasma levels of 4-kDa FITC dextran 1 h after its application.
